# Supplementary figures and images for: A mitotic chromatin phase transition prevents perforation by microtubules
Source: Nature. Author manuscript; Available in PMC 2022 Sep 21. (PMC9433320; doi:10.1038/s41586-022-05027-y)

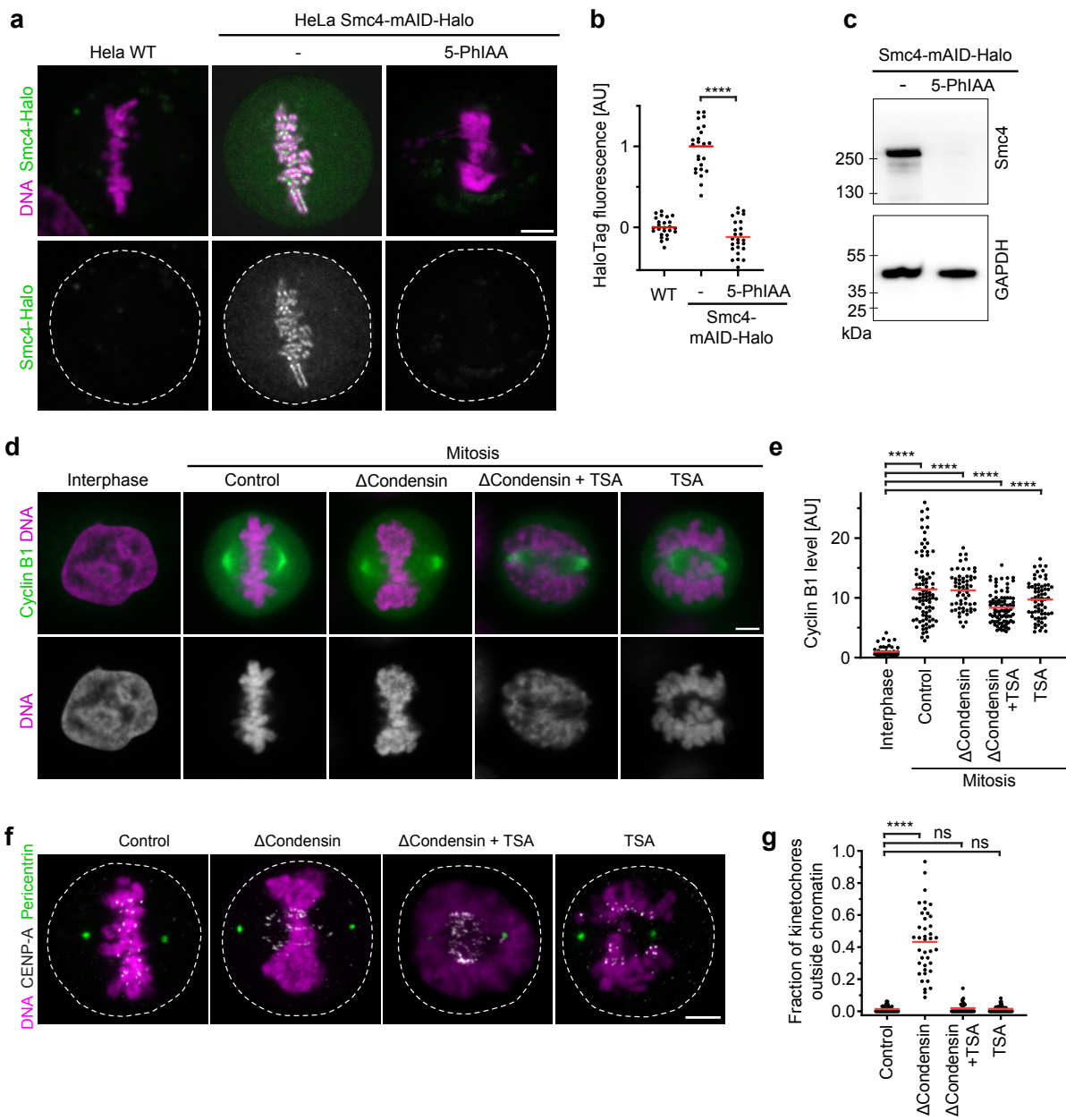

Supplement: Extended Data Fig. 1 [file EMS153637-supplement-Extended_Data_Fig__1.pdf]

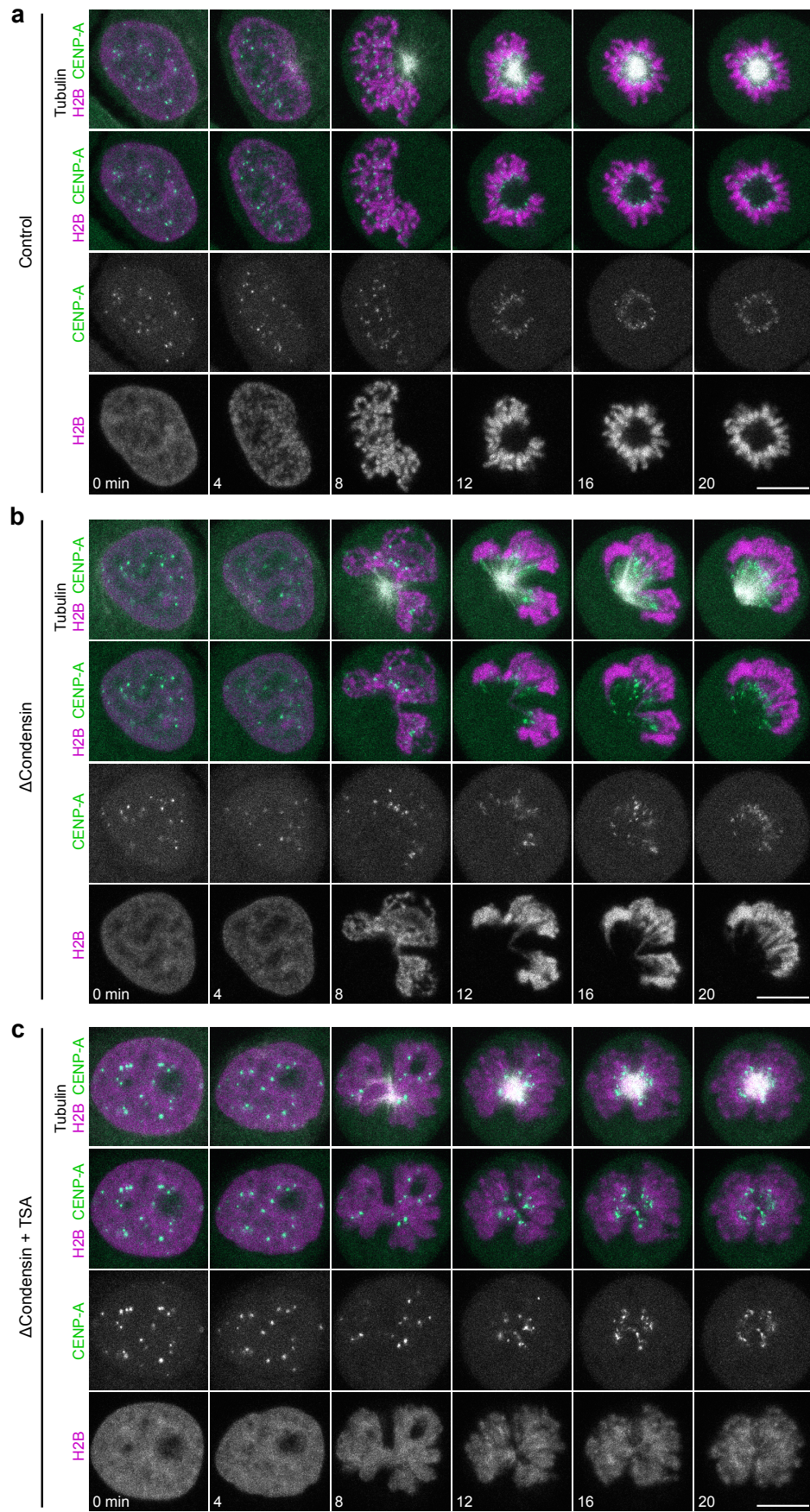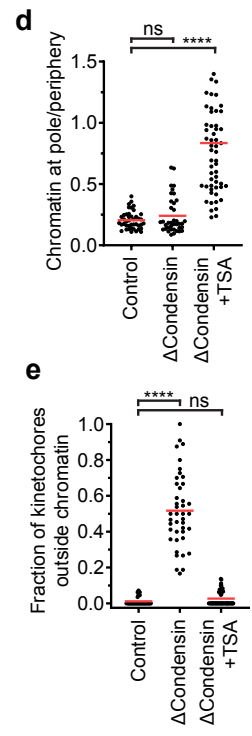

Supplement: Extended Data Fig. 2 [file EMS153637-supplement-Extended_Data_Fig__2.pdf]

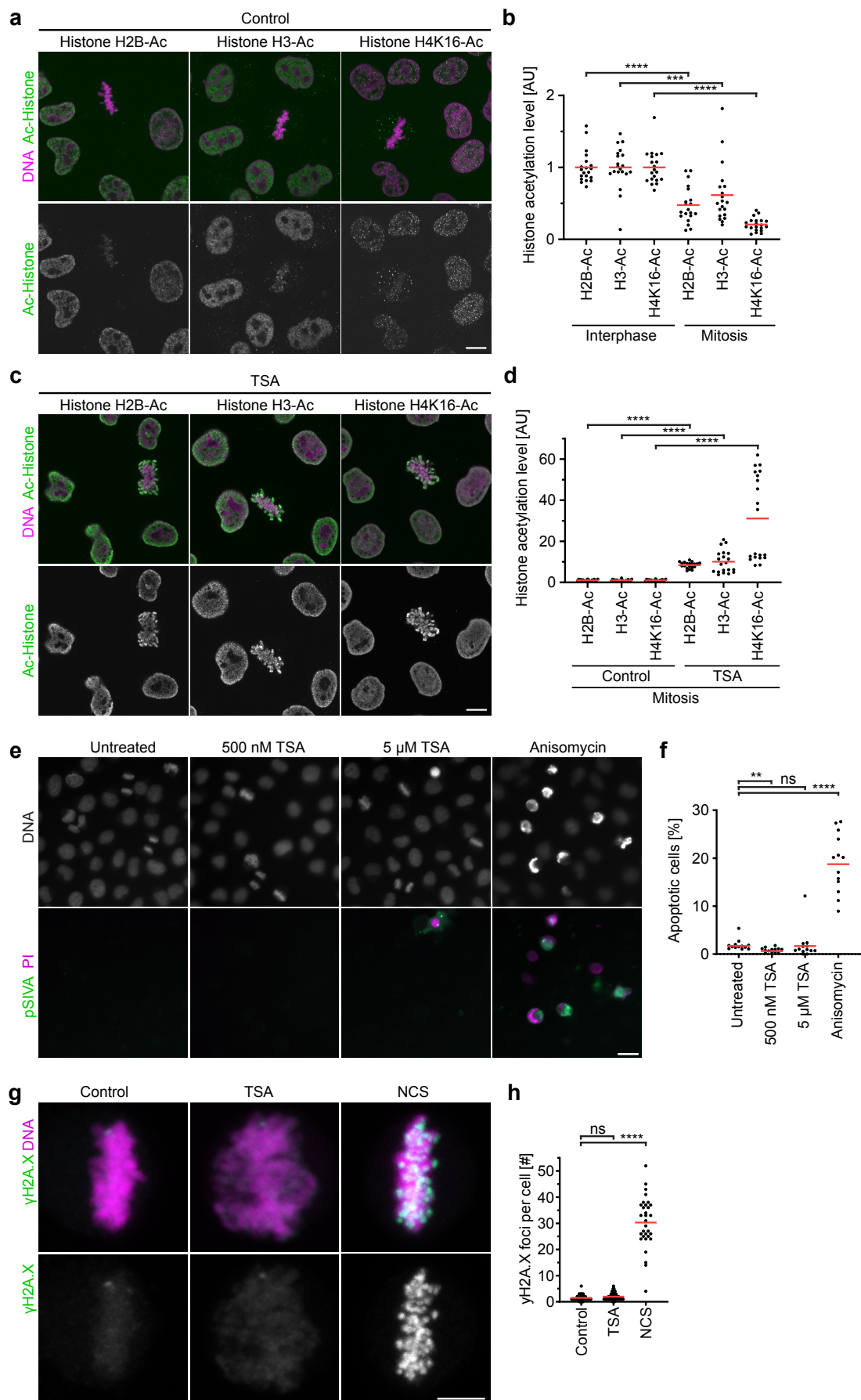

Supplement: Extended Data Fig. 3 [file EMS153637-supplement-Extended_Data_Fig__3.pdf]

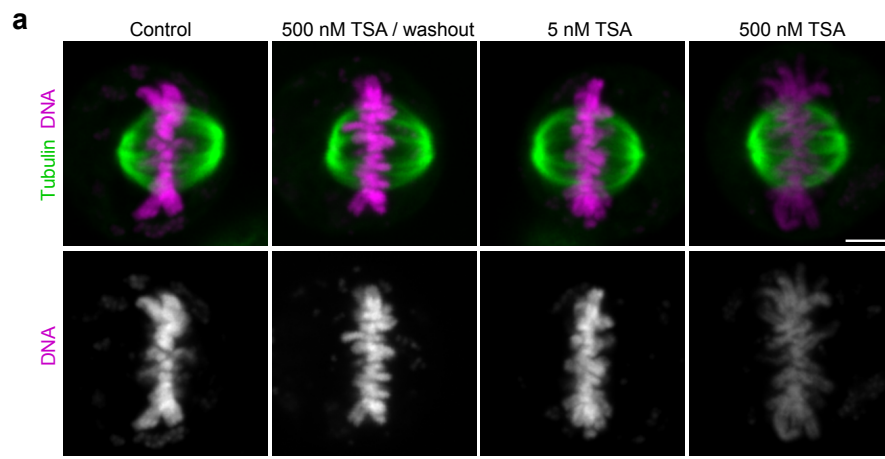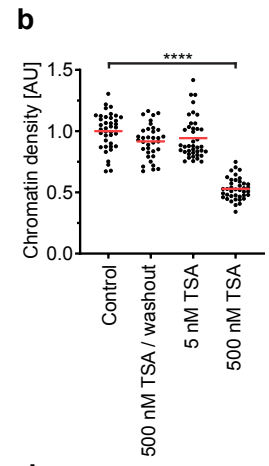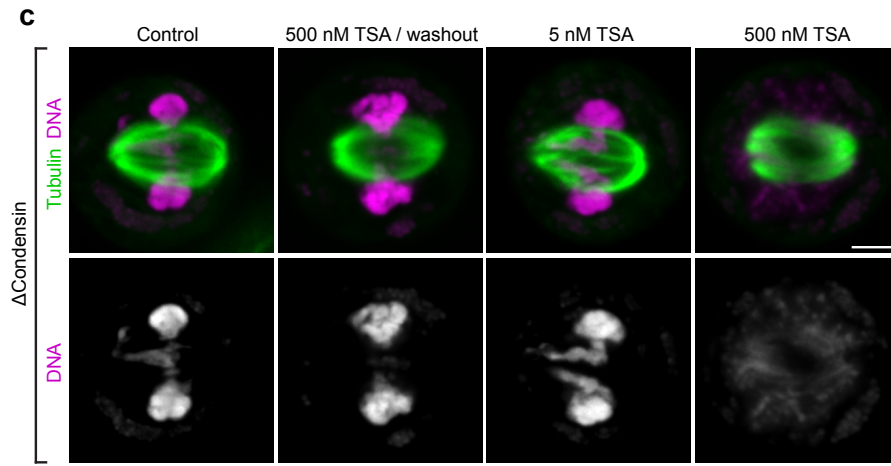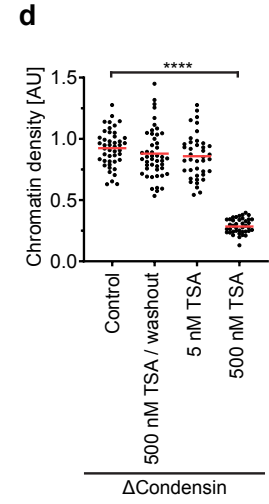

Supplement: Extended Data Fig. 4 [file EMS153637-supplement-Extended_Data_Fig__4.pdf]

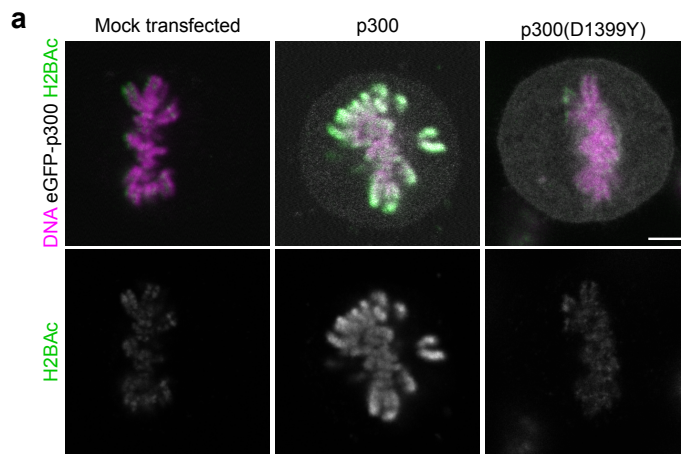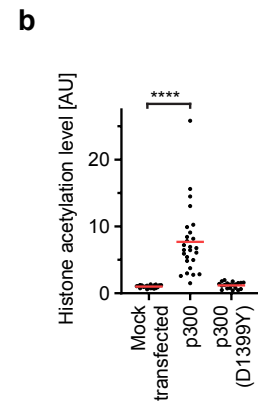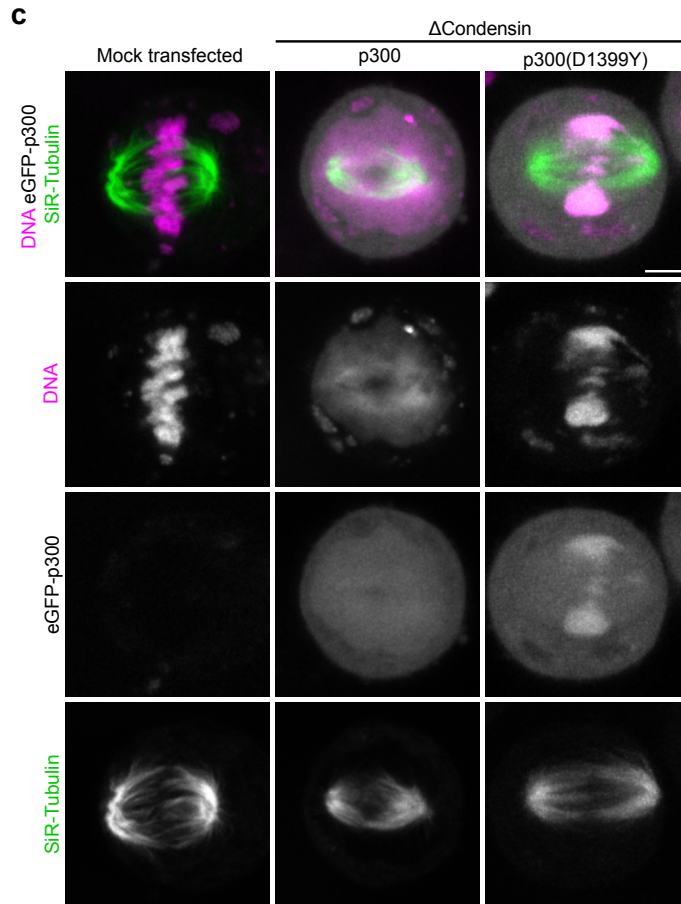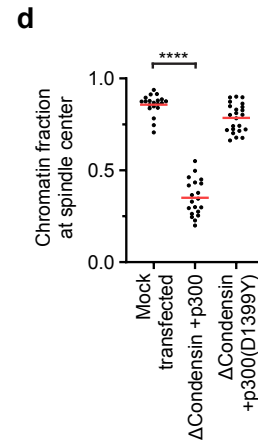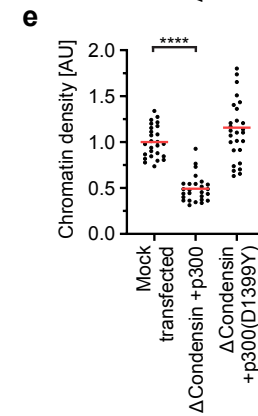

Supplement: Extended Data Fig. 5 [file EMS153637-supplement-Extended_Data_Fig__5.pdf]

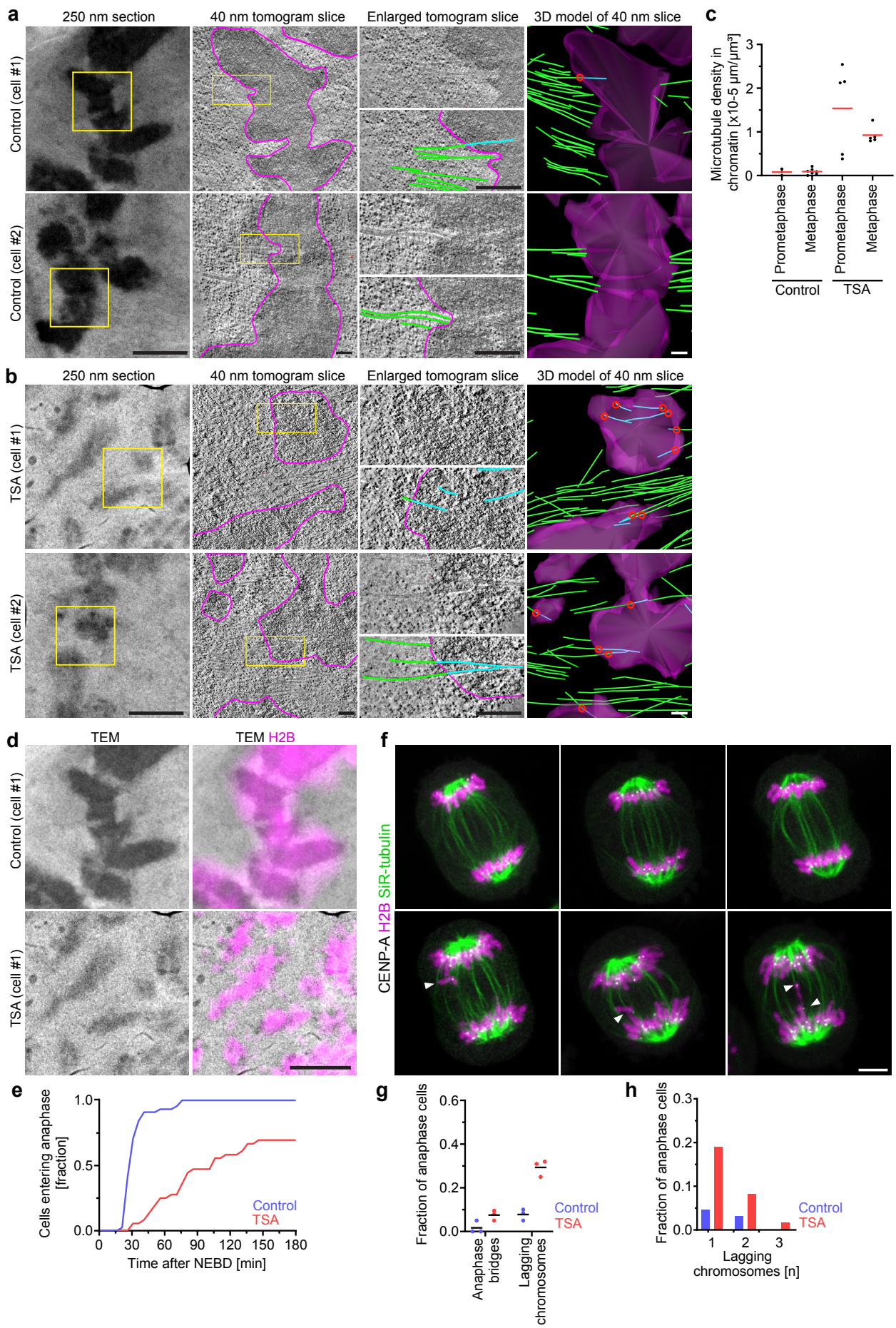

Supplement: Extended Data Fig. 6 [file EMS153637-supplement-Extended_Data_Fig__6.pdf]

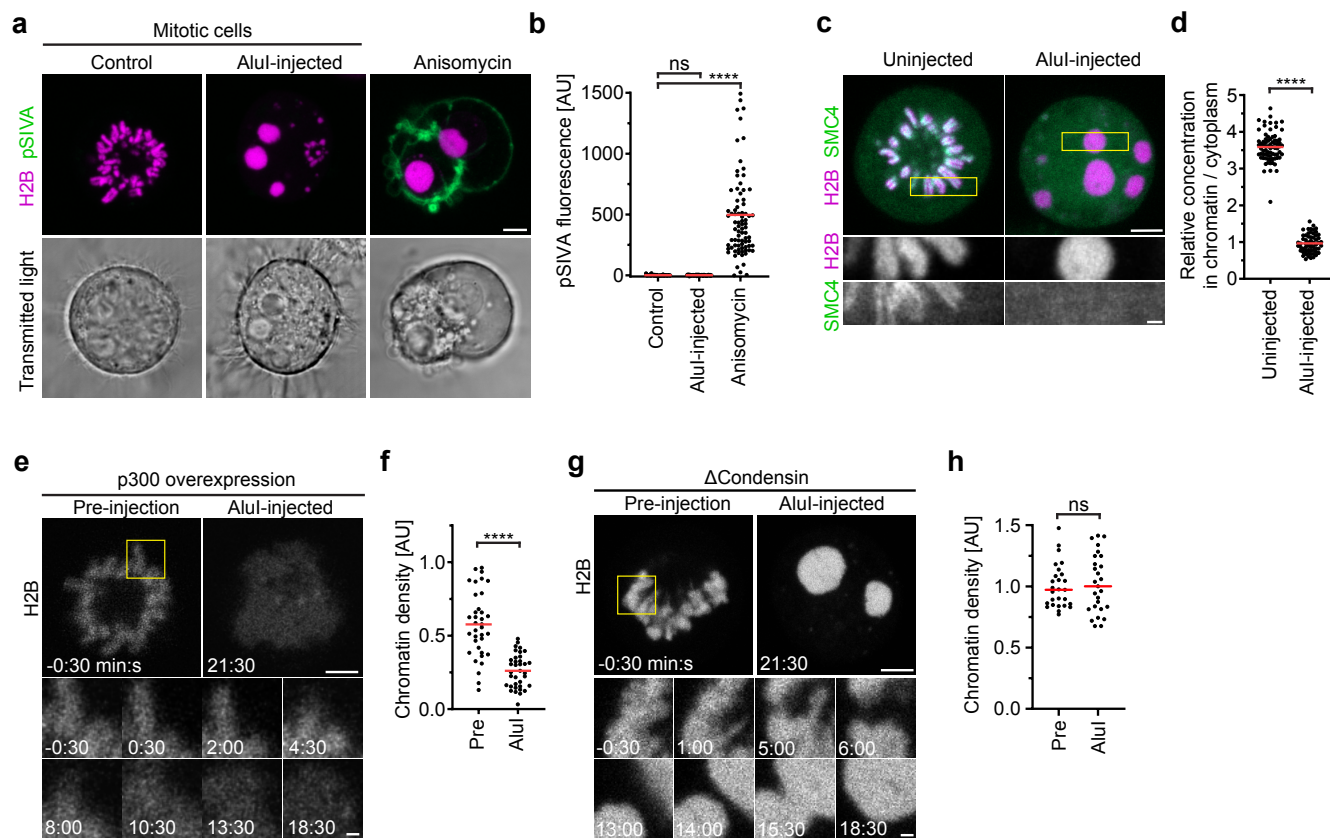

Supplement: Extended Data Fig. 7 [file EMS153637-supplement-Extended_Data_Fig__7.pdf]

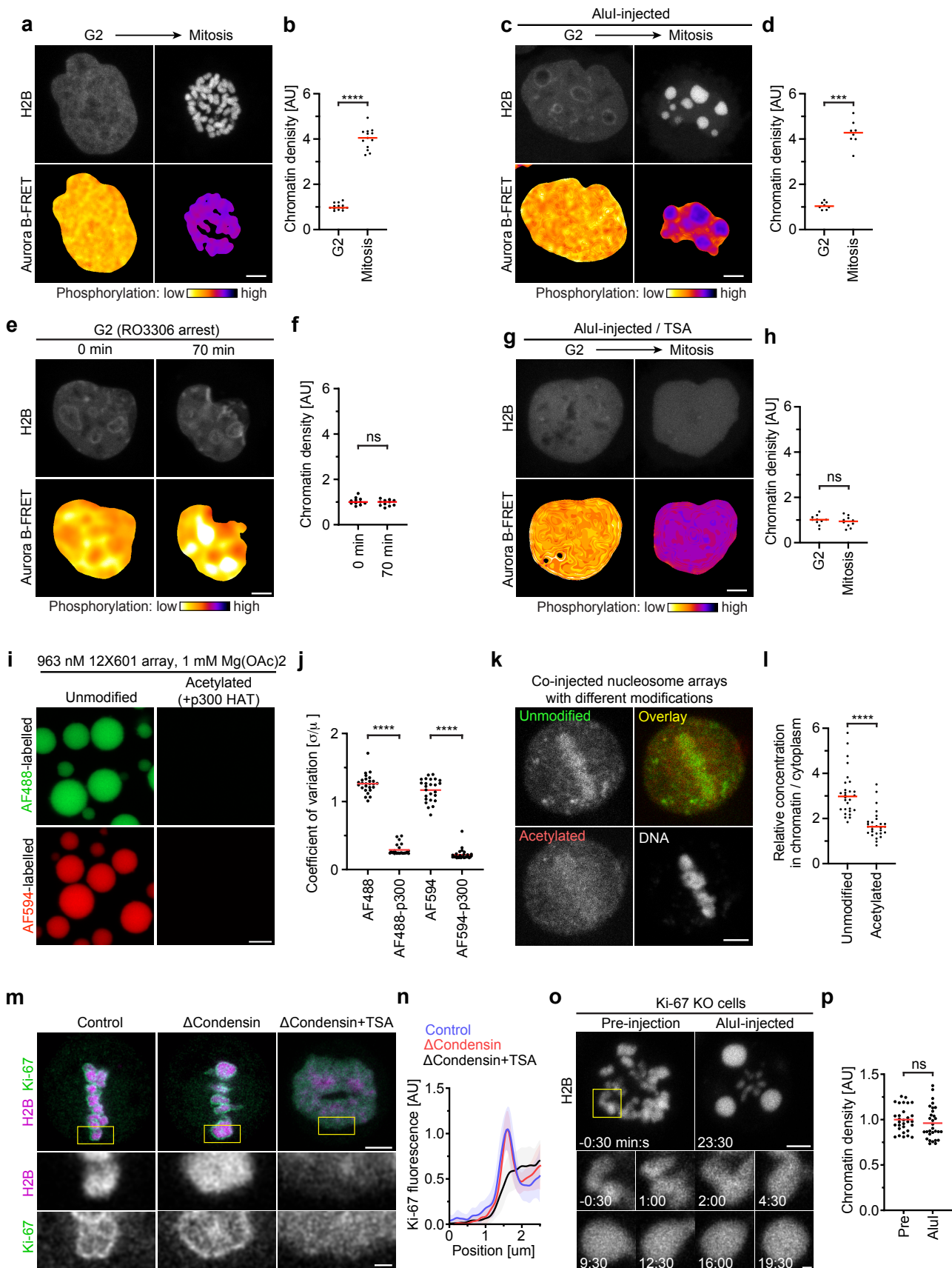

Supplement: Extended Data Fig. 8 [file EMS153637-supplement-Extended_Data_Fig__8.pdf]

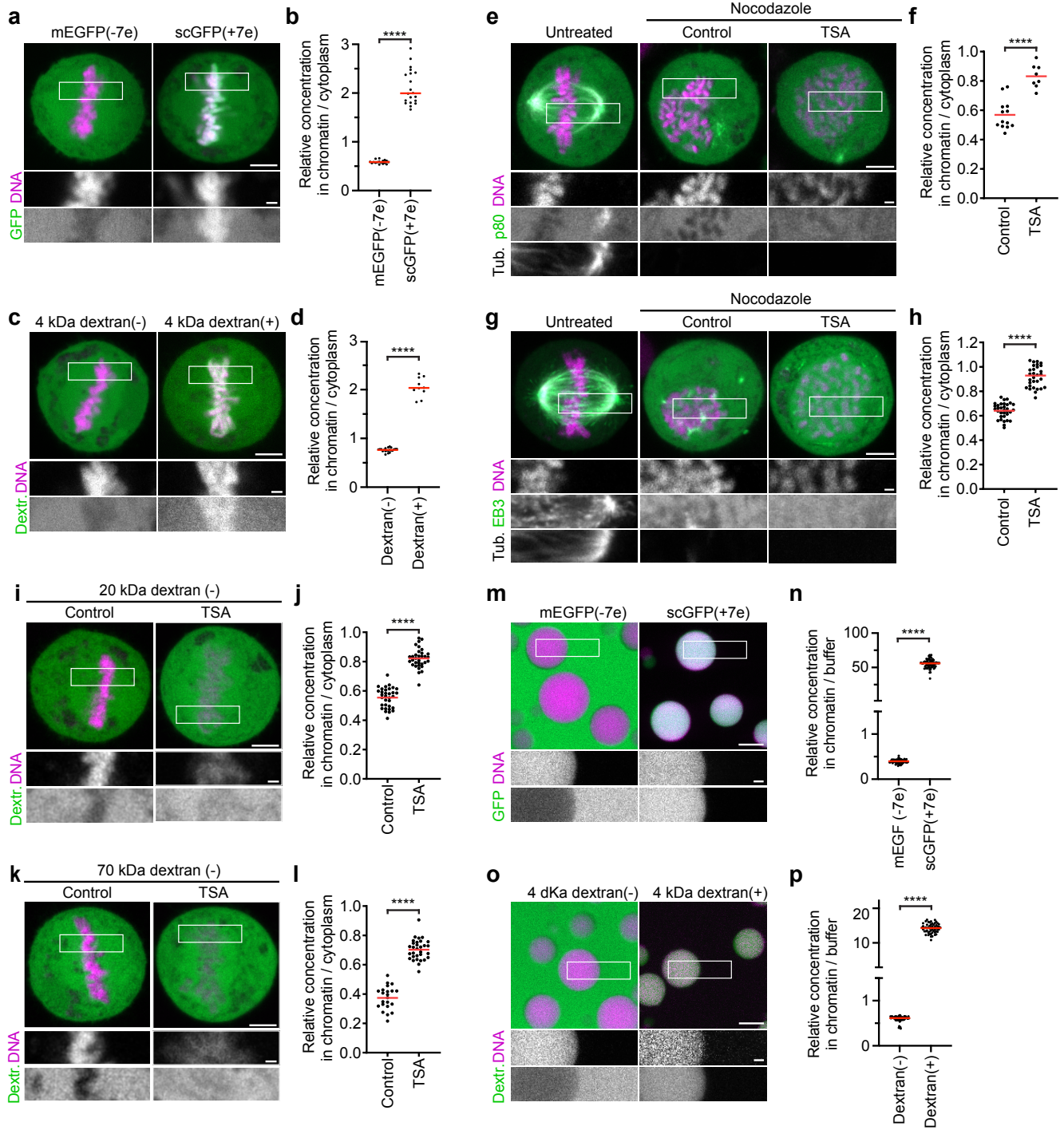

Supplement: Extended Data Fig. 9 [file EMS153637-supplement-Extended_Data_Fig__9.pdf]

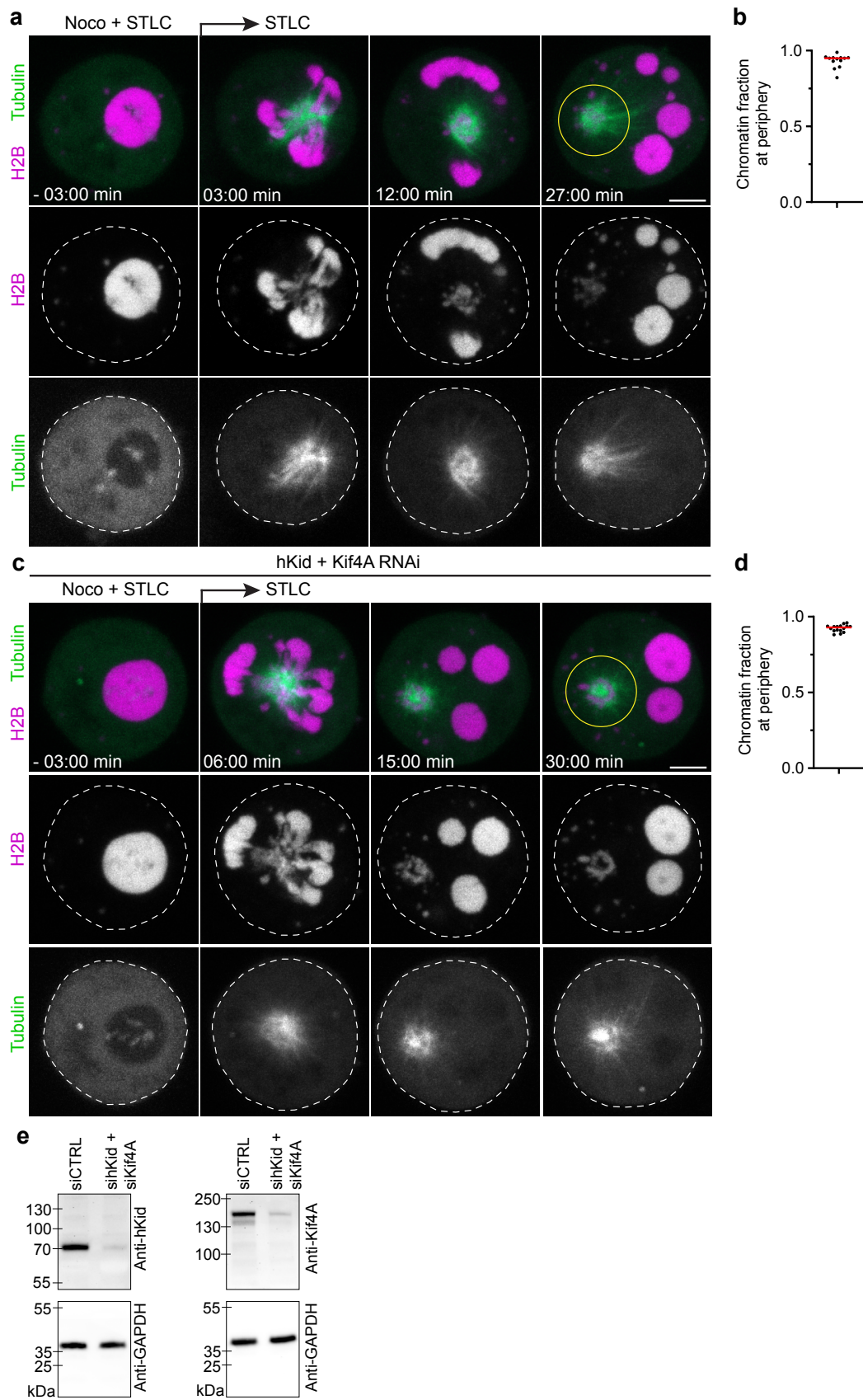

Supplement: Extended Data Fig. 10 [file EMS153637-supplement-Extended_Data_Fig__10.pdf]
