# Supplementary material for: A mitotic chromatin phase transition prevents perforation by microtubules
Source: Nature. Author manuscript; Available in PMC 2022 Sep 21. (PMC9433320; doi:10.1038/s41586-022-05027-y)
Supplement: Supplementary figure 1 [file EMS153637-supplement-Supplementary_figure_1.pdf]

nature portfolio

https://doi.org/10.1038/s41586-022-05027-y

Supplementary information

A mitotic chromatin phase transition prevents perforation by microtubules

In the format provided by the authors and unedited

a

Extended Data Fig. 1c

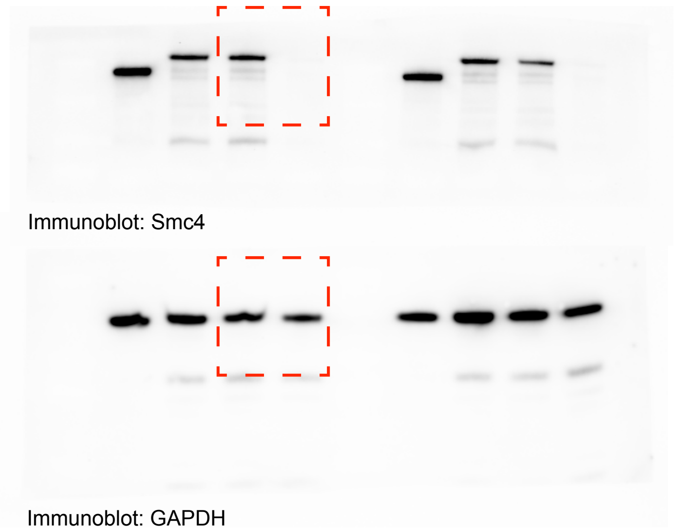

b

Extended Data Fig. 10e

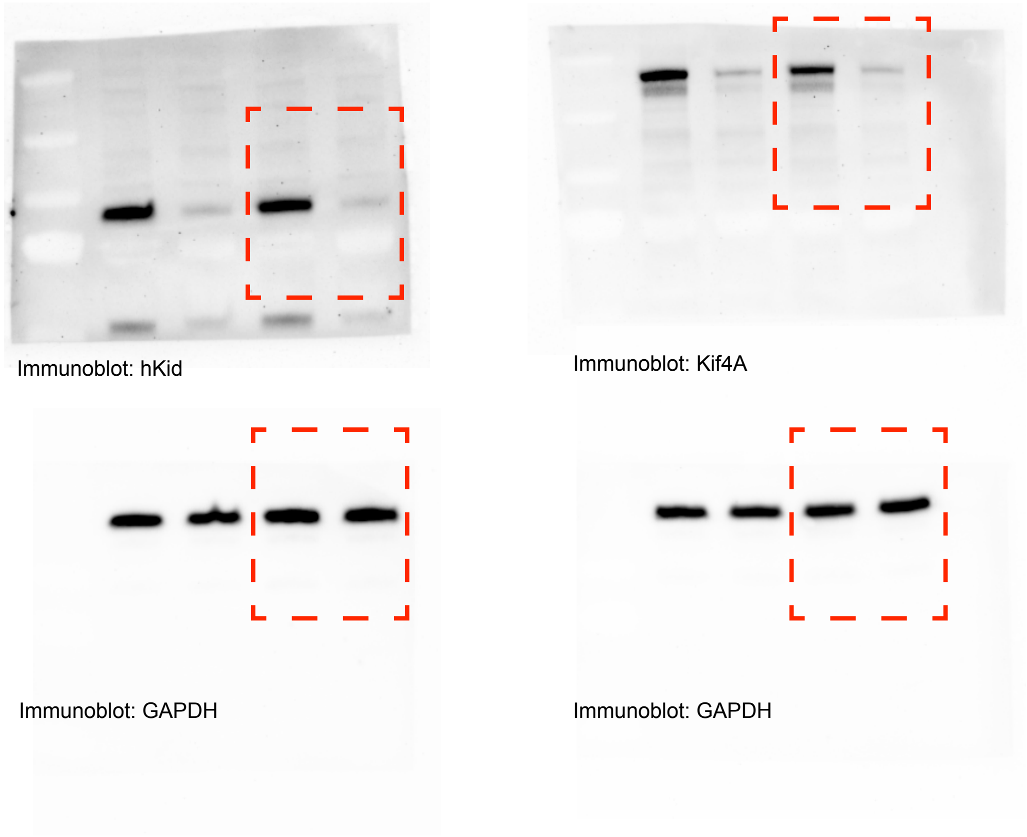

Supplementary Figure 1 | Source gel data. The full gels correspond to the data shown in **a**, Extended Data Fig. 1c or **b**, Extended Data Fig. 10e. Red boxes indicate image regions displayed in the respective figures.
