# Supplementary material for: A mitotic chromatin phase transition prevents perforation by microtubules
Source: Nature. Author manuscript; Available in PMC 2022 Sep 21. (PMC9433320; doi:10.1038/s41586-022-05027-y)
Supplement: Supplementary [file EMS153637-supplement-Supplementary.pdf]

nature portfolio

Corresponding author(s): Daniel W. Gerlich
Maximilian W.G. Schneider

Last updated by author(s): 5/24/2022

Reporting Summary

Nature Portfolio wishes to improve the reproducibility of the work that we publish. This form provides structure for consistency and transparency in reporting. For further information on Nature Portfolio policies, see our Editorial Policies and the Editorial Policy Checklist.

Statistics

For all statistical analyses, confirm that the following items are present in the figure legend, table legend, main text, or Methods section.

n/a	Confirmed	
☐	☒	The exact sample size (*n*) for each experimental group/condition, given as a discrete number and unit of measurement
☐	☒	A statement on whether measurements were taken from distinct samples or whether the same sample was measured repeatedly
☐	☒	The statistical test(s) used AND whether they are one- or two-sided<br>*Only common tests should be described solely by name; describe more complex techniques in the Methods section.*
☒	☐	A description of all covariates tested
☐	☒	A description of any assumptions or corrections, such as tests of normality and adjustment for multiple comparisons
☐	☒	A full description of the statistical parameters including central tendency (e.g. means) or other basic estimates (e.g. regression coefficient) AND variation (e.g. standard deviation) or associated estimates of uncertainty (e.g. confidence intervals)
☐	☒	For null hypothesis testing, the test statistic (e.g. *F*, *t*, *r*) with confidence intervals, effect sizes, degrees of freedom and *P* value noted<br>*Give P values as exact values whenever suitable.*
☒	☐	For Bayesian analysis, information on the choice of priors and Markov chain Monte Carlo settings
☒	☐	For hierarchical and complex designs, identification of the appropriate level for tests and full reporting of outcomes
☒	☐	Estimates of effect sizes (e.g. Cohen's *d*, Pearson's *r*), indicating how they were calculated

Our web collection on statistics for biologists contains articles on many of the points above.

Software and code

Policy information about availability of computer code

Data collection	- Images: LSM780 operated by ZEN 2011 software or LSM980 operated by ZEN Blue 2020 Software.<br>- Western blotting: BioRad Imager operated by Image Lab 6.0.1.
Data analysis	- Image analysis: ImageJ/Fiji (version 1.53c, Java 1.8.0_66 (64-bit).<br>- Plots and statistical analysis: GraphPad Prism (version 8.1.1 (330)) or Python (3.7).<br>- Electron tomogram analysis: SerialEM 4.0 and IMOD/3dmod 4.11

For manuscripts utilizing custom algorithms or software that are central to the research but not yet described in published literature, software must be made available to editors and reviewers. We strongly encourage code deposition in a community repository (e.g. GitHub). See the Nature Portfolio guidelines for submitting code & software for further information.

Data

Policy information about availability of data

All manuscripts must include a data availability statement. This statement should provide the following information, where applicable:

- Accession codes, unique identifiers, or web links for publicly available datasets
- A description of any restrictions on data availability
- For clinical datasets or third party data, please ensure that the statement adheres to our policy

All datasets generated in this study are available from the corresponding authors upon request. Source data are provided with this paper. Raw microscopy data are available and will be provided from by the corresponding authors upon request, given the large file sizes that are involved. No restrictions apply to the availability of microscopy data.

Field-specific reporting

Please select the one below that is the best fit for your research. If you are not sure, read the appropriate sections before making your selection.

☒ Life sciences ☐ Behavioural & social sciences ☐ Ecological, evolutionary & environmental sciences

For a reference copy of the document with all sections, see nature.com/documents/nr-reporting-summary-flat.pdf

Life sciences study design

All studies must disclose on these points even when the disclosure is negative.

Sample size	No sample-size calculations were performed. Sample sizes were chosen as large as possible while taking into account the experimental effort required to generate the respective data. Adequate statistics has been applied throughout the manuscript in order to make sure that the observed effects are significant given the reported sample size.
Data exclusions	These two criteria were pre-established before any analyses after data acquisition:<br>- Cells that die or move out of the field of view during movie acquisition during long time-lapse acquisitions<br>- For microinjection experiments: cells in which the plasma membrane ruptured or co-injected fluorescent tracer leaked out of the cytoplasm within <5 minutes after micro injection.
Replication	Reported experiments were repeated in at least 2 biological replicates with consistent results. Unless otherwise noted, in all analyses the biological replicates have been combined.
Randomization	Not relevant as grouping was not applied.
Blinding	To minimize potential human bias, most experiments were analyzed by prerecorded Fiji command macros (Fig. 1 a-c, 2a-i, 3a-f, ED Fig. 1a-e, ED Fig. 2a-d, 3a-h, 4a-d, 5a-e, 7a-h, 8a-l, o, p, 9a-d, m-p). When manual annotation was required, blinding precautions were made. Annotation of chromatin and microtubules in electron tomograms was performed manually (Fig. 1d-f, ED Fig. 6a-c). Ki-67 distribution was measured along line profiles drawn perpendicular to the chromatin marker surface (Fig. 2g-i), ED Fig. 8 m, n). Center of mass of spindle pole was chosen manually based on point of highest fluorescence density the respective tubulin marker (Fig. 4a, b, ED Fig. 2a-d, Fig. 10a-d). Fraction of detached kinetochores was determined manually by measuring the distance of CENP-A marker to closest chromatin marker surface (ED Fig. 1f, g, Fig. 2e). Time form NEBD to anaphase onset was manually determined in time lapse movies and fraction of lagging chromosomes was determined manually in high-resolution 3D images of anaphase cells (ED FIg. 6f-h). Partitioning coefficient of MAPs and dextrans was measured along manually defined line profiles perpendicular to the metaphase plate (ED Fig. e-l).

Reporting for specific materials, systems and methods

We require information from authors about some types of materials, experimental systems and methods used in many studies. Here, indicate whether each material, system or method listed is relevant to your study. If you are not sure if a list item applies to your research, read the appropriate section before selecting a response.

Materials & experimental systems

n/a	Involved in the study
☐	☒ Antibodies
☐	☒ Eukaryotic cell lines
☒	☐ Palaeontology and archaeology
☒	☐ Animals and other organisms
☒	☐ Human research participants
☒	☐ Clinical data
☒	☐ Dual use research of concern

Methods

n/a	Involved in the study
☒	☐ ChIP-seq
☒	☐ Flow cytometry
☒	☐ MRI-based neuroimaging

Antibodies

Antibodies used	Primary antibodies:<br>- anti-CENP-A (ENZO Life Sciences, clone 3-19, ADI-KAM-CC006-E, 10161910), dilution 1:1000<br>- Pericentrin (Abcam, clone EPR21987, ab220784, GR3284309-1), dilution 1:2000<br>- Acetylated histone 2B (Millipore, 07-373, 3092508), dilution 1:500<br>- Acetylated histone 3 (Merck, 06-599, 3260200), dilution 1:500<br>- Acetylated histone 4K16 (Abcam, clone EPR1004, ab109463, GR284778-8), dilution 1:500<br>- γH2A.X (BioLegend, clone 2F3, 613402, B283251), dilution 1:1000<br>- Cyclin B1 (Cell Signaling, clone D5C10, 12231S, 7), dilution 1:800<br>- Smc4 (Abcam, ab229213, GR3228108-5), dilution 1:1000<br>- GAPDH (Abcam, ab9485, GR3212164-2), dilution 1:2500<br>- hKid/Kif22 (Abcam, clone EP2747Y, ab75783, GR129278-4), dilution 1:1000

- Kif4a (Abcam, clone EPR5459, ab124903, GR96215-7), dilution 1:1000

Secondary antibodies:
- goat anti-mouse Alexa Fluor 488 (Molecular Probes, A11001, 1787787), dilution 1:1000
- goat anti-rabbit Alexa Fluor 633 (Molecular Probes, A21071, 99E2-1), dilution 1:1000
- donkey anti-rabbit Alexa Fluor 488 (Molecular Probes, A21206, 1796375), dilution 1:1000
- horseradish peroxidase goat anti-mouse (Biorad, cat. number 1706516), dilution 1:5000
- horseradish peroxidase goat anti-rabbit (Biorad, cat. number 1706515), dilution 1:5000

Validation

anti-CENP-A antibody was validated by immunoblotting using an appropriate molecular weigth marker in Jurkat and Raji cell lines. References: Gene replacement strategies validate the use of functional tags on centromeric chromatin and invalidate an essential role for CENP-AK124ub: C. Salinas-Luypaert, et al.; Cell Rep. 37, 10924 (2021), Human Artificial Chromosomes that Bypass Centromeric DNA: G.A. Logsdon, et al.; Cell 178, 624 (2019), Phosphorylation of CENP-A on serine 7 does not control centromere function: V. Barra, et al.; Nat. Commun. 10, 175 (2019), CENP-A Modifications on Ser68 and Lys124 Are Dispensable for Establishment, Maintenance, and Long-Term Function of Human Centromeres: D. Fachinetti, et al.; Dev. Cell 40, 104 (2017), Centromeres are maintained by fastening CENP-A to DNA and directing an arginine anchor-dependent nucleosome transition: L.Y. Guo, et al.; Nat. Commun. 8, 15775 (2017).
anti-Pericentrin antibody was validated by IP and immunoblotting usign appropriate molecular weigth markers, intracellular flow cytometry analysis and immunofluorescence staining in HepG2, NIH/3T3 and HeLa cell lines. References: Vergarajauregui S et al. AKAP6 orchestrates the nuclear envelope microtubule-organizing center by linking golgi and nucleus via AKAP9. Elife 9:N/A (2020).
anti-acetylated histone 2B antibody was validated using immunoblot with appropriate molecular weigth markers using extract from HeLa cells with an extract of sodium butyrate treated cells as positive control.
anti-acetylated histone 3 antibody was validated by immunoblot using appropriate molecular weigth markers in extracts of Hela cells with sodium butyrate treated cells as positive control and recombinant histone 3 as a negative control.
anti-acetylated histone 4K16 antibody was validated using immunoblot with appropriate molecular weigth markers using extract from HeLa and C6 cell lines and mouse spleen lysate, with TSA treated cells as positive control, by immunofluorescence in HeLa cells with TSA treated cells as a positive control and by intracellular flow cytometry staining in HeLa cells. References (limited to the last 2 years): Song Z et al. Effects of histone H4 hyperacetylation on inhibiting MMP2 and MMP9 in human amniotic epithelial cells and in premature rupture of fetal membranes. Exp Ther Med 21:515 (2021), Shalini V et al. Genome-wide occupancy reveals the localization of H1T2 (H1fnt) to repeat regions and a subset of transcriptionally active chromatin domains in rat spermatids. Epigenetics Chromatin 14:3 (2021), Contreras SM et al. Resveratrol induces H3 and H4K16 deacetylation and H2A.X phosphorylation in Toxoplasma gondii. BMC Res Notes 14:19 (2021), Huang R et al. HDAC11 inhibition disrupts porcine oocyte meiosis via regulating a-tubulin acetylation and histone modifications. Aging (Albany NY) 13:8849-8864 (2021), Navarro-Carrasco E & Lazo PA VRK1 Depletion Facilitates the Synthetic Lethality of Temozolomide and Olaparib in Glioblastoma Cells. Front Cell Dev Biol 9:683038 (2021), Sui L et al. HDAC11 promotes meiotic apparatus assembly during mouse oocyte maturation via decreasing H4K16 and a-tubulin acetylation. Cell Cycle 19:354-362 (2020), Du L et al. Loss of SIRT4 promotes the self-renewal of Breast Cancer Stem Cells. Theranostics 10:9458-9476 (2020), Sun X et al. Histone deacetylase inhibitor valproic acid attenuates high glucose-induced endoplasmic reticulum stress and apoptosis in NRK-52E cells. Mol Med Rep 22:4041-4047 (2020), Koziol K et al. Changes in ?H2AX and H4K16ac levels are involved in the biochemical response to a competitive soccer match in adolescent players. Sci Rep 10:14481 (2020), Kubatka P et al. Rhus coriaria L. (Sumac) Demonstrates Oncostatic Activity in the Therapeutic and Preventive Model of Breast Carcinoma. Int J Mol Sci 22:N/A (2020).
anti-yH2A.X antibody was validated by immunoblot using appropriate molecular weigth markers, with extract from HeLa cells, using extract from UV-treated cells as positive control and by immunofluorescence of HeLa cells using UV treated cells as a positive control. Application references: Akbay A, et al. 2008. Am J Pathol. 173:536. (IHC), Mochizuki K, et al.2008.J cell Sci.121:2148. (IF), Xiao R, et al. 2007. Mol Cell Biol.27:5393. (IF), Rossi DJ, et al. 2007. Nature. 447:725. (IF), Loidl J, et al. 2009. Mol Cell Biol. 20:2048. (IF), Beels L, et al. 2009. Circulation. 120:1903. (IF), Yamada C, et al. 2010 J. Biol. Chem. 285:16693. (WB), Bu Y, et al. 2010, Biochem Biophys Res Commun. 397:157. (WB), Massignan T, et al. 2010. J. Biol Chem. 285:7752. (WB).
anti-CyclinB1 antibody was validated by immunoblot using appropriate molecular weigth markers in extracts from HT-29, HeLa, Jurkat and HCT 116 cells and in extracts from synchronized HT-29 cells following thymidine block and release for various times, indicating cell cycle dependent chagnes in Cyclin B1 levels as well as immunofluorescence in HT-29 cells. REferences: Zhang J, Li A, Sun H, Xiong X, Qin S, Wang P, Dai L, Zhang Z, Li X, Liu Z. Amentoflavone triggers cell cycle G2/M arrest by interfering with microtubule dynamics and inducing DNA damage in SKOV3 cells. Oncol Lett. 2020 Nov;20(5):168. doi: 10.3892/ol.2020.12031. Epub 2020 Aug 27. PMID: 32934735; PMCID: PMC7471765, Lee CAA, Banerjee P, Wilson BJ, Wu S, Guo Q, Berg G, Karpova S, Mishra A, Lian JW, Tran J, Emmerich M, Murphy GF, Frank MH, Frank NY. Targeting the ABC transporter ABCB5 sensitizes glioblastoma to temozolomide-induced apoptosis through a cell-cycle checkpoint regulation mechanism. J Biol Chem. 2020 May 29;295(22):7774-7788. doi: 10.1074/jbc.RA120.013778. Epub 2020 Apr 20. PMID: 32317280; PMCID: PMC7261782., Zheng Z, Wu M, Zhang J, Fu W, Xu N, Lao Y, Lin L, Xu H. The Natural Compound Neobractatin Induces Cell Cycle Arrest by Regulating E2F1 and Gadd45α. Front Oncol. 2019 Jul 17;9:654. doi: 10.3389/fonc.2019.00654. PMID: 31380287; PMCID: PMC6653061., Zheng Z, Wu M, Zhang J, Fu W, Xu N, Lao Y, Lin L, Xu H. The Natural Compound Neobractatin Induces Cell Cycle Arrest by Regulating E2F1 and Gadd45α. Front Oncol. 2019 Jul 17;9:654. doi: 10.3389/fonc.2019.00654. PMID: 31380287; PMCID: PMC6653061, Tan X, Yuan G, Wang Y, Zou Y, Luo S, Han H, Qin Z, Liu Z, Zhou F, Liu Y, Yao K. RAB20 Promotes Proliferation via G2/M Phase through the Chk1/cdc25c/cdc2-cyclinB1 Pathway in Penile Squamous Cell Carcinoma. Cancers (Basel). 2022 Feb 22;14(5):1106. doi: 10.3390/cancers14051106. PMID: 35267417; PMCID: PMC8909501, Zhang X, Zhao J, Gao X, Pei D, Gao C. Anthelmintic drug albendazole arrests human gastric cancer cells at the mitotic phase and induces apoptosis. Exp Ther Med. 2017 Feb;13(2):595-603. doi: 10.3892/etm.2016.3992. Epub 2016 Dec 22. PMID: 28352336; PMCID: PMC5348670.
anti-Smc4 antibody was validated by immunoblot using appropriate molecular weigth markers using extracts from HEK-293T, A431, HeLa and HepG2 cells. We verified the molecular weight of the target protein by immunoblotting with extracts from HeLa cells by using appropriate molecular weight range markers. 5-PhIAA treatment caused loss of the band of Smc4-mAID-HaloTag.
anti-GAPDH antibody was validated by immunoblot using appropriate molecular weigth markers in lysate from Hela, Jurkat, A431, HEK-293 and HepG2 cells, by immunofluorescence in HeLa cells and by ELISA using extracts from human fibroblast. References: Ni W et al. Preventing oxaliplatin-induced neuropathic pain: Using berberine to inhibit the activation of NF-?B and release of pro-inflammatory cytokines in dorsal root ganglions in rats. Exp Ther Med 21:135 (2021), Ge R et al. Upregulated microRNA-126 induces apoptosis of dental pulp stem cell via mediating PTEN-regulated Akt activation. J Clin Lab Anal 35:e23624 (2021), Zhang Y et al. Long Non-coding RNA CASC15 Promotes Intrahepatic Cholangiocarcinoma Possibly through Inducing PRDX2/PI3K/AKT Axis. Cancer Res Treat 53:184-198 (2021), Reed JC et al. Identification of an Antiretroviral Small Molecule That Appears To Be a Host-Targeting Inhibitor of HIV-1 Assembly. J Virol 95:N/A (2021), Yao W et al. TNK2-AS1 upregulated by YY1 boosts the course of osteosarcoma

through targeting miR-4319/WDR1. Cancer Sci 112:893-905 (2021), Zhang Z et al. lncRNA CASC9 sponges miR-758-3p to promote proliferation and EMT in bladder cancer by upregulating TGF-ß2. Oncol Rep 45:265-277 (2021).
anti-hKid/Kif22 antibody was validated by immunoblot using appropriate molecular weigth markers in extracts from wild-type and knockout HEK-293T cells and extracts from HeLa cells. We verified the molecular weigth of the target protein in extracts from HeLa cells by immunoblotting following RNAi which led to loss of the band. References: Li C et al. NuSAP governs chromosome oscillation by facilitating the Kid-generated polar ejection force. Nat Commun 7:10597 (2016).
anti-Kif4A antibody was validated by immunoblot using appropriate molecular weigth controls in mouse thymus tissue lysate and extracts from HeLa and HEK293T cells as well as by intracellular immunofluorescence followed by flow cytometry in HEK293T cells. We verified the molecular weigth of the target protein in extracts from HeLa cells by immunoblotting following RNAi which led to loss of the band.

Eukaryotic cell lines

Policy information about cell lines

Cell line source(s)	All cells lines were derived from a HeLa cell line ("Kyoto" strain) obtained from S. Narumiya (Kyoto University, Japan). The original commercial source of HeLa cells is the American Type Culture Collection (ATCC).
Authentication	Wild-type HeLa Kyoto cells were validated by a Multiplex human Cell line Authentication test (MCA), 21.04.16.
Mycoplasma contamination	Routine mycoplasma tested showed all cell lines were free of mycoplasma contamination.
Commonly misidentified lines (See ICLAC register)	HeLa cells are not in the list of commonly misidentified cell lines.
